# Serological evidence of possible high levels of undetected transmission of Zika virus among Papua New Guinea military personnel, 2019

**DOI:** 10.1016/j.ijregi.2022.07.006

**Published:** 2022-07-16

**Authors:** Richard Grant, Joanne Kizu, Melissa Graham, Fiona McCallum, Brady McPherson, Alyson Auliff, Peter Kaminiel, Wenjun Liu

**Affiliations:** a Arbovirology Department, Australian Defence Force Malaria and Infectious Disease Institute, Enoggera, Brisbane, Queensland, Australia; b Queensland Institute of Medical Research–Berghofer Medical Research Institute, Brisbane, Queensland, Australia; c Operational Health, Joint Health Command, Canberra, Australian Capital Territory, Australia; d Health Services, Papua New Guinea Defence Force, Port Moresby, Papua New Guinea

**Keywords:** Antibody, Arbovirus, Papua New Guinea military personnel, Seroprevalence, Zika virus

## Abstract

**Objectives:**

The Papua New Guinea (PNG) Health Department retrospectively reported six cases of Zika virus (ZIKV) from a cohort of febrile patients during outbreaks of dengue and malaria in 2016. However, the transmission of ZIKV remains unclear due to lack of testing capability. This study aimed to determine the level of immunity to ZIKV among PNG military personnel (PNGMP) in 2019.

**Methods:**

Sera of 208 PNGMP recruited in April 2019 was tested for the presence of anti-ZIKV immunoglobulin G (IgG) and M (IgM) antibodies using Euroimmun IgG/IgM detection kits, and anti-ZIKV neutralizing antibody (Nab) against a ZIKV African strain on all anti-ZIKV-IgG/IgM^+^ samples.

**Results:**

Anti-ZIKV seropositivity of these sera was as follows: IgG, 67%; IgM, 9%; and Nab, 65%. Five of 19 anti-ZIKV-IgM^+^ samples had anti-ZIKV-Nab titres ≥20, as well as an anti-ZIKV-Nab titre ratio ≥4 compared with the Nab titres of four anti-dengue serotypes, so met the criteria of the World Health Organization (WHO) for confirmed ZIKV infection.

**Conclusions:**

The prevalence of anti-ZIKV-Nab of 65% suggests that there are high levels of ZIKV exposure among PNGMP. Five of the 19 anti-ZIKV-IgM^+^ samples met the WHO criteria for confirmed ZIKV infection, suggesting a recent undetected outbreak in PNGMP. These results provide better understanding of the current ZIKV epidemic status in PNGMP.

## Highlights

• In total, 208 Papua New Guinea military personnel (PNGMP) participated in this survey.
• The overall seroprevalence of Zika virus IgG using indirect enzyme-linked immunosorbent assay was 67%.
• The overall seroprevalence of Zika virus by neutralizing assay was 65%.
• Five of 19 anti-ZIKV-IgM^+^ samples met the criteria of the World Health Organization for confirmed ZIKV infection.
• An undetected Zika virus outbreak may have occurred in PNGMP in 2019.

## Introduction

Zika virus (ZIKV) infection is associated with congenital neurological abnormalities, such as fetal microcephaly and Guillain–Barré syndrome (Baker et al., 2022). ZIKV infection can cause an acute febrile illness with symptoms of fever, rash, joint pain and conjunctivitis, which may be misdiagnosed as malaria, dengue or chikungunya in co-circulation regions (Haby et al., 2018). Retrospective testing of samples collected from febrile patients during a dengue and malaria outbreak in Papua New Guinea (PNG) in 2014–2016 identified six patients infected with ZIKV with no history of travel outside of PNG (World Health Organization, 2016).

Due to a lack of testing capability, the actual incidence of ZIKV infection in PNG remains largely unknown. Currently, laboratory diagnosis of ZIKV infection depends on the detection of anti-ZIKV-IgM antibody, which normally appears in serum 5–7 days after disease onset. The authors conducted a population-based ZIKV seroprevalence survey on sera obtained from PNG military personnel (PNGMP) in April 2019 to determine the level of immunity to ZIKV among PNGMP.

## Methods

These results are part of an infectious disease surveillance study conducted by the Australian Defence Force in conjunction with the PNG Defence Force in April 2019. Seventy-six PNGMP from Manus Island and 132 PNGMP from Wewak consented voluntarily to participate in this survey. All participants were asked if they had experienced febrile illness and travelled within the 4 weeks preceding blood sampling (Table 1).

**Table 1:** Clinical and serological observations for 208 Papua New Guinea military personnel participating in this study

Military participants	Manus Island Barracks	Wewak Barracks	Total
Number of participants	76	132	208
Percentage	36.4	63.6	100%
Male/female	76/0	131/1	207/1
Age range (years)^a^	23–62	20–59	20–62
Mean	35.2	39.2	37.5
Median	29	41.5	34
Travel history in 4 weeks preceding blood draw date			
In PNG	39.5% (30/76)	34.1% (45/132)	36.2% (75/208)
Overseas	0%	0%	0%
Anti-ZIKV ELISA (reactive/tested number)			
IgG^+^	69.7% (53/76)	66% (87/132)	67.3% (140/208)
IgM^+^	9% (7/76)	9.1% (12/132)	9.1% (19/208)
Total positivity of ELISA IgG+IgM	72.3% (55/76)	68.9% (92/132)	70.7% (147/208)
Both ELISA IgG^+^ and ELISA IgM^+^	6.6% (5/76)	5.3% (7/132)	5.8% (12/208)
Anti-ZIKV neutralization antibody in ELISA-reactive samples	63.2% (48/76)	65.9% (87/132)	64.9% (135/208)
Anti-ZIKV IgM^+^ soldiers with clinical symptoms (fever/chills, cough ≥2 weeks, body aches) in 4 weeks preceding blood draw	57.1% (4/7)	91.7 (11/12)	78.9% (15/19)
IgM^+^ soldiers treated with antimalarial/antibiotic drugs	14.3% (1/7)	41.7% (5/12)	31.6%(6/19)
Neutralization positivity (MR766) of ELISA IgM^+^ samples	100% (7/7)	83.3% (10/12)	89.5% (17/19)

ZIKV, Zika virus; ELISA, enzyme-linked immunosorbent assay; Ig, immunoglobulin.

a Age = blood draw date - date of birth.

Anti-ZIKV, anti-Japanese encephalitis virus (JEV) and anti-dengue NS1 protein specific IgG/IgM antibodies were detected using enzyme-linked immunosorbent assay (ELISA) kits. Anti-ZIKV ELISA^+^ samples were tested for anti-ZIKV neutralizing antibody (Nab) against the prototype strain MR766 (Rhesus/1947/Uganda/MR766). Anti-ZIKV-IgM^+^ samples were also tested for anti-dengue Nabs with strains of dengue-1_Hawaii_, dengue-2_NGC_, dengue-3_H-87_ and denuge-4_H-241_. Detailed methods are described in Appendix 1 (see online supplementary material).

## Results

The prevalence rates of anti-ZIKV were as follows: IgG, 67%; IgM, 9%; IgG+IgM, 71%; and Nab, 65% (Table 1). The anti-ZIKV-Nab titre ranged from 10 to ≥640 (data not shown). Seventeen of 19 anti-ZIKV-IgM^+^ subjects had anti-ZIKV-Nab to the MR766 strain (Table 2). Among the anti-ZIKV-IgM^+^Nab^−^ samples, Sample 215 was also anti-dengue-IgM^+^ and Sample 155 was also anti-JEV-IgM^+^, indicating that these two samples could be false anti-ZIKV-IgM^+^ due to cross-reactivity with dengue virus or JEV. All anti-ZIKV-IgM^+^ samples had detectable anti-dengue-Nabs to multiple serotypes (Table 2). Five samples (Nos 39, 66, 88, 138 and 165) had anti-ZIKV-Nab titres ≥20 and a Nab titre ratio ≥4 compared with the anti-dengue 4 serotype Nab titres (Table 2), which met the criteria of the World Health Organization (WHO) for confirmed ZIKV infectiion (World Health Organization, 2019). Fifteen of 19 anti-ZIKV-IgM^+^ PNGMP reported clinical symptoms within the 4 weeks preceding blood sampling (Table 1), with six of them also reporting antibiotic and antimalarial treatment, highlighting the possibility of undetected ZIKV infection. Nine anti-ZIKV-Nab^−^ control samples, including one sample diagnosed previously with dengue 3 and one sample from a dengue-vaccinated individual, did not neutralize ZIKV.

**Table 2:** Anti-Zika virus (ZIKV), dengue virus and Japanese encephalitis virus (JEV) antibody profiles for 19 Papua New Guinea military personnel who were anti-ZIKA IgM^+^

Serum no., age (years)	ELISA antibody						Neutralization antibody titre				
	ZIKV		Dengue		JEV		Dengue				ZIKV
	IgM	IgG	IgM	IgG	IgM	IgG	Dengue 1	Dengue 2	Dengue 3	Dengue 4	MR766
39, 28	+	+	-	+	+	+	40	20	80	20	320
41, 28	+	-	-	+	-	+	80	0	40	10	10
60, 30	+	+	-	+	-	+	80	40	320	40	320
62, 25	+	+	-	+	-	+	160	160	640	20	640
63, 58	+	-	-	+	-	+	80	40	40	10	160
66, 33	+	+	-	+	-	+	10	10	40	0	640
71, 45	+	+	-	+	-	+	160	80	40	10	160
88, 48	+	+	+	+	+	+	20	20	20	20	320
96, 32	+	-	-	+	+	+	160	80	20	20	20
100, 34	+	-	-	+	-	+	160	40	20	20	40
138, 48	+	+	-	+	-	+	40	40	40	10	160
139, 46	+	+	-	+	+	+	80	160	640	40	10
155, 32	+	-	+	+	-	+	80	20	80	20	0
157, 51	+	+	-	+	-	+	80	20	160	40	10
160, 50	+	-	-	+	-	+	80	80	320	40	160
165, 31	+	+	-	+	+	+	0	10	0	10	320
207, 30	+	-	-	+	-	+	320	80	80	40	40
208, 28	+	+	-	+	+	+	80	160	320	40	40
215, 37	+	+	-	+	+	+	80	40	160	20	0

ELISA, enzyme-linked immunosorbent assay.

## Discussion and conclusions

Anti-ZIKV-IgM typically develops by the end of the first week after symptom onset, and remains detectable for approximately 3 months (Griffin et al., 2019). The present data, showing that five PNGMP met the WHO criteria for recent ZIKV infection, and a prevalence of anti-ZIKV-Nab of 65% in PNGMP, suggest that there may have been significant, undetected transmission of ZIKV in PNGMP recently.

Seroprevalence rates of approximately 71% for anti-ZIKV ELISA IgG/M antibodies and approximately 65% for anti-ZIKV-Nabs among PNGMP are comparable with rates in French Polynesia (66%, ELISA IgG) (Aubry et al., 2017) and Yap Island, Micronesia (73%, ELISA IgG/IgM) (Duffy et al., 2009) where ZIKV is endemic. It remains unclear why ZIKV-related microcephaly has not been reported in PNG despite the high seroprevalence of antibodies in PNGMP. The shortage of reliable diagnostic, reporting and monitoring systems that track virus transmission may be a partial explanation. High endemicity of dengue in PNG may also be a contributor, as a high level of multi-type dengue virus antibody and anti-dengue CD8+ T cells may be protective against congenital ZIKV syndrome (Wen et al., 2017; Pedroso et al., 2019). Another hypothesis is that phenotypic changes in Asian lineage ZIKV strains may have led to differing disease outcomes (Weaver et al., 2016). Differences in ZIKV exposures between PNGMP and the general population may also be a factor.

The higher proportion of samples showing anti-ZIKV-IgG positivity compared with anti-ZIKV-Nab positivity could be due to the endemicity of other flavivirus that are antigenically closely related to ZIKV, such as dengue virus and JEV. The finding of two samples that were anti-ZIKV-IgM^+^ but anti-ZIKA-Nab^−^ indicates that the current commercially available ELISA detection kits for ZIKV may not be suitable for diagnostic or seroprevalence survey purposes in dengue- and JEV-endemic areas, such as PNG, due to possible serological cross-reactivity among flaviviruses (Maeki et al., 2019; Montecillo-Aguado et al., 2019) (Table 2). All samples that tested anti-ZIKV-IgG/IgM^+^ on ELISA should be confirmed by neutralizing assay for diagnostic/surveillance purposes.

This preliminary finding requires further support from additional investigations, as the present study had a small sample size and only PNGMP were included. The authors intend to expand their arbovirus surveillance programme in PNG to include investigation of circulating ZIKV strains, mosquito behaviours and antibody prevalence amongst the entire population of PNG in order to better understand the potential risk of ZIKV transmission in PNG.

## Appendix Supplementary materials

Image, application 1

## References

[bib0001] Aubry M, Teissier A, Huart M, Merceron S, Vanhomwegen J, Roche C (2017). Zika virus seroprevalence, French Polynesia, 2014–2015. Emerg Infect Dis.

[bib0002] Baker RE, Mahmud AS, Miller IF, Rajeev M, Rasambainarivo F, Rice BL (2022). Infectious disease in an era of global change. Nat Rev Microbiol.

[bib0003] Duffy MR, Chen TH, Hancock WT, Powers AM, Kool JL, Lanciotti RS (2009). Zika virus outbreak on Yap Island, Federated States of Micronesia. N Engl J Med.

[bib0004] Griffin I, Martin SW, Fischer M, Chambers TV, Kosoy O, Falise A (2019). Zika virus IgM detection and neutralizing antibody profiles 12–19 months after illness onset. Emerg Infect Dis.

[bib0005] Haby MM, Pinart M, Elias V, Reveiz L. (2018). Prevalence of asymptomatic Zika virus infection: a systematic review. Bull World Health Organ.

[bib0006] Maeki T, Tajima S, Ikeda M, Kato F, Taniguchi S, Nakayama E (2019). Analysis of cross-reactivity between flaviviruses with sera of patients with Japanese encephalitis showed the importance of neutralization tests for the diagnosis of Japanese encephalitis. J Infect Chemother.

[bib0007] Montecillo-Aguado MR, Montes-Gomez AE, Garcia-Cordero J, Corzo-Gomez J, Vivanco-Cid H, Mellado-Sanchez G (2019). Cross-reaction, enhancement, and neutralization activity of dengue virus antibodies against Zika virus: a study in the Mexican Population. J Immunol Res.

[bib0008] Pedroso C, Fischer C, Feldmann M, Sarno M, Luz E, Moreira-Soto A (2019). Cross-protection of dengue virus infection against congenital Zika syndrome, Northeastern Brazil. Emerg Infect Dis.

[bib0009] Weaver SC, Costa F, Garcia-Blanco MA, Ko AI, Ribeiro GS, Saade G (2016). Zika virus: history, emergence, biology, and prospects for control. Antiviral Res.

[bib0010] Wen J, Elong Ngono A, Regla-Nava JA, Kim K, Gorman MJ, Diamond MS (2017). Dengue virus-reactive CD8(+) T cells mediate cross-protection against subsequent Zika virus challenge. Nat Commun.

[bib0011] World Health Organization (2016). https://www.who.int/emergencies/disease-outbreak-news/item/22-april-2016-zika-png-en.

[bib0012] World Health Organization. Geneva: WHO. 2019. Available at: https://www.who.int/publications/i/item/WHO-ZIKV-LAB-16.1.

