# Supplementary material for: Serological evidence of possible high levels of undetected transmission of Zika virus among Papua New Guinea military personnel, 2019
Source: IJID Reg. 2022 Jul 16;4:131–3. doi: 10.1016/j.ijregi.2022.07.006 (PMC9340500; doi:10.1016/j.ijregi.2022.07.006)
Supplement: Supplementary file 1 [file mmc1.docx]

Appendix

ELISAs

Commercially available Dengue ELISA kits from Abbott, Australia (Abbott, https://www.globalpointofcare.abbott/en/index.html), Zika virus (ZIKV) and Japan encephalitis virus (JEV) ELISA kits Euroimmun (Euroimmun, https://www.euroimmun.com) were used according to the manufacturer’s protocol for DENV ELISA IgG (product code 01PE30), IgM (product code SD01PE20), ZIKV IgG (product code 2668-9601G) and IgM (product code EI 2668-9601M), JEV IgG (EI2663-9601 G) and IgM (EI2663-9601 M). Positivity was determined by comparing the sample result to the IgM and IgG reference sera provided (cut-off calibrators). A positive sample was defined as having a sample/calibrator absorbance ratio of ≥1.1, and a negative sample was defined as having a ratio of <1.1.

**Micro-neutralization assay:**

ZIKV specific neutralizing antibody responses were assessed using a micro-neutralization assay (MN) on Vero cells as ZIKV infection causes clear cytopathic effects (CPE) (Sherman et al., 2019), performed according to the methods described in publication for ZIKV(Nascimento et al., 2019). Briefly, Vero cells were seeded into 96-well plates at a density of 1.5 × 10^4^ cells per well one day before the assay. Sera samples to be tested were diluted starting from 1:10 with dilution medium (DM) (RPMI supplemented with 2% foetal bovine serum, 10 mM HEPES, 20 mM L-glutamine, 100 units/mL of penicillin and 100 μg/mL, all above reagents were purchased from Life Technology). Two-fold serial dilutions (1:10, 1:20, 1:40, 1:80, 1:160, 1:320, and 640) in duplicate were performed using 60 μL aliquots across rows A-G rows a 96 well plate. To wells G1-G6 of the plate were added 60 μL DM only rather than testing sera as virus control and wells G7-G12 wells were used as cell only controls. Sixty μL of virus stock containing 120 TCID50 infectious dose of ZIKV virus were mixed with the two-fold serially diluted sera in all test wells and incubated at 37°C with 5% v/v CO 2 / air for 1 hour to neutralize the virus. To the cell control wells, 120μL of DM was added only. Following neutralization, 100 μL of sera-virus mixtures were added into the 96 well plates to infect the Vero cells. The plates were incubated at 37°C in an atmosphere of 5% v/v CO_2_/ air except all plates were gently shaking at 30rmp/min for 30 minutes outside the incubator every day after infection. Six days post infection, cells were fixed with 3.7% formaldehyde, stained with 1% crystal violet for 1 hour, washed in tap water, dried and the absorbance (O.D.) of wells were read at 595nm wavelength using a 96 well plate spectrophotometer. The O.D. of virus control wells in the same plate must be lower than 0.3 and the O.D. of the cell control wells at least 1.5. The cut-off value used to determine neutralizing antibody was calculated according to following formula: Cut off value=(mean O.D. of 6 virus control wells of in the same plate+ mean O.D. of 6 cell control wells in the same plate)/2. All values above the cut off value were considered positive for neutralization antibody. The reciprocal antibody dilution corresponding to that well is the 50% neutralization antibody titre for that human sample. Anti-ZIKV neutralizing antibody was reported as a reciprocal titre and the specific MN titres of ≥10 were considered positive.

DENV specific neutralizing antibody was determined on C6-36 cells using a standard DENV immuno-ELISA assays (indirect ELISA) as DENV infection does not cause clear CPE on C6-36 or Vero cells as described before (Pickering, Hugo, Devine, Aaskov, & Liu, 2020). Briefly, monolayers of C6-36 cells in 96 well plates were infected with serum-virus mixture described as above. Six days after infection, cell monolayers were fixed by ice cold methanol: acetone at ratio of 1:1 at 4°C for 1 hour, then cells were stained with cocktail monoclonal antibodies 4G2:6B6C1:2H2 at 1:1:1, followed by horseradish peroxidase-conjugated rabbit anti-mouse secondary antibodies (Dako, Cat. No.P0260, Denmark). After washing the cell monolayers with 0.5% v/v Tween 20 (Merck, USA)/PBS, plates were visualised by adding 3,3,5,5 tetramethylbenzidine (TMB; Sigma, USA). Mock-infected cultures, maintained in parallel, were used as controls. Cell staining was visualised using an inverted microscope and virus infected cells were stained as blue colour. The endpoint titration of absence of infection (approaching the colour of mock-infected control wells) was considered the neutralizing titre. Anti-dengue neutralizing antibody was reported as a reciprocal titre and the specific MN titres of ≥10 were considered positive.

**References:**

Nascimento, E. J. M., Bonaparte, M. I., Luo, P., Vincent, T. S., Hu, B., George, J. K., . . . Huleatt, J. W. (2019). Use of a Blockade-of-Binding ELISA and Microneutralization Assay to Evaluate Zika Virus Serostatus in Dengue-Endemic Areas. *Am J Trop Med Hyg, 101*(3), 708-715. doi:10.4269/ajtmh.19-0270

Pickering, P., Hugo, L. E., Devine, G. J., Aaskov, J. G., & Liu, W. (2020). Australian Aedes aegypti mosquitoes are susceptible to infection with a highly divergent and sylvatic strain of dengue virus type 2 but are unlikely to transmit it. *Parasit Vectors, 13*(1), 240. doi:10.1186/s13071-020-04091-5

Sherman, K. E., Rouster, S. D., Kong, L. X., Aliota, M. T., Blackard, J. T., & Dean, G. E. (2019). Zika virus replication and cytopathic effects in liver cells. *PLoS One, 14*(3), e0214016. doi:10.1371/journal.pone.0214016
